# The effects of demographic, social, and environmental characteristics on pathogen prevalence in wild felids across a gradient of urbanization

**DOI:** 10.1371/journal.pone.0187035

**Published:** 2017-11-09

**Authors:** Jesse S. Lewis, Kenneth A. Logan, Mat W. Alldredge, Scott Carver, Sarah N. Bevins, Michael Lappin, Sue VandeWoude, Kevin R. Crooks

**Affiliations:** 1 Department of Fish, Wildlife, and Conservation Biology, Graduate Degree Program in Ecology, Colorado State University, Fort Collins, CO, United States of America; 2 Mammals Research, Colorado Parks and Wildlife, Montrose, CO, United States of America; 3 Mammals Research, Colorado Parks and Wildlife, Fort Collins, CO, United States of America; 4 School of Biological Sciences, University of Tasmania, Hobart, Tasmania, Australia; 5 USDA-APHIS-Wildlife Services’ National Wildlife Research Center, Fort Collins, CO, United States of America; 6 Department of Clinical Sciences, Colorado State University, Fort Collins, CO, United States of America; 7 Department of Microbiology, Immunology, and Pathology, Colorado State University, Fort Collins, CO, United States of America; Universidade de Aveiro, PORTUGAL

## Abstract

Transmission of pathogens among animals is influenced by demographic, social, and environmental factors. Anthropogenic alteration of landscapes can impact patterns of disease dynamics in wildlife populations, increasing the potential for spillover and spread of emerging infectious diseases in wildlife, human, and domestic animal populations. We evaluated the effects of multiple ecological mechanisms on patterns of pathogen exposure in animal populations. Specifically, we evaluated how ecological factors affected the prevalence of *Toxoplasma gondii* (Toxoplasma), *Bartonella spp*. (Bartonella), feline immunodeficiency virus (FIV), and feline calicivirus (FCV) in bobcat and puma populations across wildland-urban interface (WUI), low-density exurban development, and wildland habitat on the Western Slope (WS) and Front Range (FR) of Colorado during 2009–2011. Samples were collected from 37 bobcats and 29 pumas on the WS and FR. As predicted, age appeared to be positively related to the exposure to pathogens that are both environmentally transmitted (Toxoplasma) and directly transmitted between animals (FIV). In addition, WS bobcats appeared more likely to be exposed to Toxoplasma with increasing intraspecific space-use overlap. However, counter to our predictions, exposure to directly-transmitted pathogens (FCV and FIV) was more likely with decreasing space-use overlap (FCV: WS bobcats) and potential intraspecific contacts (FIV: FR pumas). Environmental factors, including urbanization and landscape covariates, were generally unsupported in our models. This study is an approximation of how pathogens can be evaluated in relation to demographic, social, and environmental factors to understand pathogen exposure in wild animal populations.

## Introduction

Infectious diseases play important roles in wildlife conservation and can threaten species and populations across local to global scales [1–6]. By modifying landscapes and altering wildlife communities, humans can influence patterns of disease dynamics in wildlife populations [1, 7–9], increasing the potential for spillover and spread of emerging infectious diseases in wildlife, human, and domestic animal populations [1, 10–12]. A primary driver of landscape alteration is urbanization. The conversion of natural areas to human development, including residences, buildings, and roads, is one of the most extensive anthropogenic disturbances affecting wildlife populations globally [13, 14] and urbanization is projected to increase by millions of hectares over the next few decades [15–17]. To conserve animal populations and reduce the risk of infectious and zoonotic pathogen spread in wildlife and humans, it is critical to understand the mechanisms that affect patterns of disease in wildlife populations across different forms of urbanization, particularly as it relates to modes of pathogen transmission [18–21].

Transmission of pathogens among animals is influenced by demographic, social, and environmental factors [7, 22, 23]. With regard to demography, males and older individuals often exhibit a greater prevalence of parasites and disease [24–28]. In many mammals, males tend to have larger extents of space use [29] and greater potential for contacts among animals [30]. In addition, because larger extents of space use allow animals a greater opportunity to interact with the landscape, animals can potentially experience increased exposure to pathogens in the environment. Increases in population density can result in both higher contact rates [31] and greater prevalence and diversity of pathogens among individuals [22]. In contrast, transmission of parasites within populations also can decrease with increasing host density, associated with less mixing among individuals within a population and more localized disease transmission [32].

Social organization plays an important role in disease transmission through intra- and interspecific interactions and contact patterns [22, 33]. For many solitary species (such as many carnivores), intraspecific social interactions primarily occur during the mating season or when defending and maintaining territorial boundaries [34]. In addition, space-use overlap of animals can lead to kleptoparasitism (where one animal steals the food of another) [35, 36], aggressive encounters, and intraguild predation [37, 38], which are behaviors that can increase the opportunity for pathogen transmission through direct and indirect interactions [4, 39]. Interspecific interactions can be important determinants of pathogen spillover from a reservoir species to another species and in some cases this has led to population decline and extirpation [4, 40, 41]. Although social organization is often associated with direct contacts between animals, it can also impact indirect contacts. Overlap in space use and maintaining territorial boundaries through marking behavior can influence indirect disease transmission pathways for animals using shared areas through environmental contamination [42].

Lastly, the environment plays a critical role in disease transmission. Landscape characteristics, including habitat features, geographic barriers, and anthropogenic factors, can influence the spread and occurrence of pathogens [43–48], and land-use change can have important implications for the distribution and abundance of pathogens [49]. Urbanization can alter the environmental conditions that influence the transmission and prevalence of pathogens through modifying landscape patterns [18, 48, 50–53] and disease spillover [54]. Pathogens originating from anthropogenic sources can increase in prevalence in animal populations associated with urbanized environments [55]. Although population densities for some species can be substantially higher in urban environments compared to rural areas, the number of infected individuals and burden of disease vectors can be substantially greater in rural populations as a result of the population ecology of intermediate hosts [56–59]. For example, agricultural areas can experience high prevalence of Toxoplasma due to abundant small mammals acting as intermediate hosts [60].

Carnivores harbor a suite of pathogens, which can impact predator populations, ecological communities, and human health [2]. In addition, pathogen exposure can vary by anthropogenic factors, such as in bobcats (*Lynx rufus*) and pumas (*Puma concolor*) [47, 48, 61], which share a broad geographic distribution in western North America. Cross-species transmission of species-specific pathogens has been reported between bobcats and pumas in highly urbanized landscapes, potentially as a result of increased contacts and aggressive encounters resulting from elevated space-use overlap within habitat fragments [62]. In addition, domestic cats (both pet and feral populations) associated with human residences harbor a suite of pathogens that can be transmitted to and from wild felids in rural and urbanized environments [54, 61, 63, 64]. Previous research evaluating pathogens in wild and domestic felids across California, Florida, and Colorado found that several demographic, social, and environmental factors were important determinants of exposure to multiple pathogens at broad scales [48, 61]. However, analyses across finer scales are necessary to understand the effects of these three factors at the individual level, particularly related to space use and social interactions within and between species.

Our goal was to investigate how multiple mechanisms influence seroprevalence of pathogens in medium and large-sized carnivores persisting across a gradient of urbanization. We evaluated bobcat and puma populations across wildland-urban interface (WUI), low-density exurban development, and wildland habitat in relation to four common pathogens in felids: *Toxoplasma gondii* (Toxoplasma), *Bartonella spp*.(Bartonella), feline immunodeficiency virus (FIV), and feline calicivirus (FCV). We predicted that (1) pathogens acquired primarily through prey and the environment (i.e., Toxoplasma) would be associated with suitable habitat for the pathogen and greater amounts of space use sharing among felids; (2) pathogens transmitted by flea vectors (i.e., Bartonella) would be associated with habitat that harbored fleas and increased social interactions (i.e., space-use overlap and number of potential contacts); and (3) pathogens that are directly transmitted between individuals (i.e., FIV and FCV) would be positively related to social interactions (Table 1). In addition, owing to potential associations with domestic cats, we expected that animals associated with habitat modified by urbanization (exurban and wildland-urban interface) would exhibit greater prevalence of pathogens shared between domestic and wild felids, compared to wild felids within wildland areas (Table 1). Consistent with previous research, we also expected that older individuals and males would be more likely to be exposed to pathogens [48, 61].

**Table 1:** Predictions of how demographic, social, and environmental characteristics will influence exposure of pathogens in bobcat and puma populations. For each pathogen, the transmission model is included in parentheses. For each factor (demographic, social, and environment), the expected relative effect strength of each prediction is included in parentheses.

Pathogen	Demographic	Social	Environment
*Toxoplasma gondii* (Consuming infected intermediate host or acquiring oocysts from environment)	1. Higher prevalence in males and older animals (strong). 2. Higher prevalence as space-use extent increases due to interacting with more of the landscape (moderate).	1. Increased space-use overlap (both intra- and interspecific) increases oocyst presence in environment leading to greater prevalence in prey and increasing opportunity to be infected through environmental contamination (moderate).	1. Animals with more NDVI in their extent of space use will be more likely to be infected (moderate). 2. Greater prevalence in areas of low-density residential development (strong).
*Bartonella spp*. (Vector-borne)	1. Higher prevalence in older animals (strong). 2. Higher prevalence as space-use extent increases due to interacting with more of the landscape (moderate).	1. Increased opportunities for intraspecific interactions leads to greater opportunity to transmit fleas (moderate).	1. Animals with more NDVI in their extent of space use will be more likely to be infected (moderate). 2. Greater prevalence in areas of low-density residential and WUI development (strong).
Feline Immunodeficiency Virus (FIV) (Direct contact)	1. Higher prevalence in males and older animals (strong). 2. Higher prevalence as space-use extent increases due to interacting with more individuals (moderate).	1. Increased opportunities for intraspecific interactions in both felids (strong) and interspecific interactions for pumas (weak) increases prevalence.	1. Greater exposure is expected in urbanized areas due to increased interactions (strong).
Feline Calicivirus (FCV) (Direct contact)	1. Higher prevalence in males and older animals (strong). 2. Higher prevalence as space-use extent increases due to interacting with more individuals (moderate).	1. Increased opportunities for intra- and interspecific interactions increases prevalence (strong).	1. Greater exposure is expected in urbanized areas due to increased interactions (strong).

## Materials and methods

### Study area

**Fig 1:**
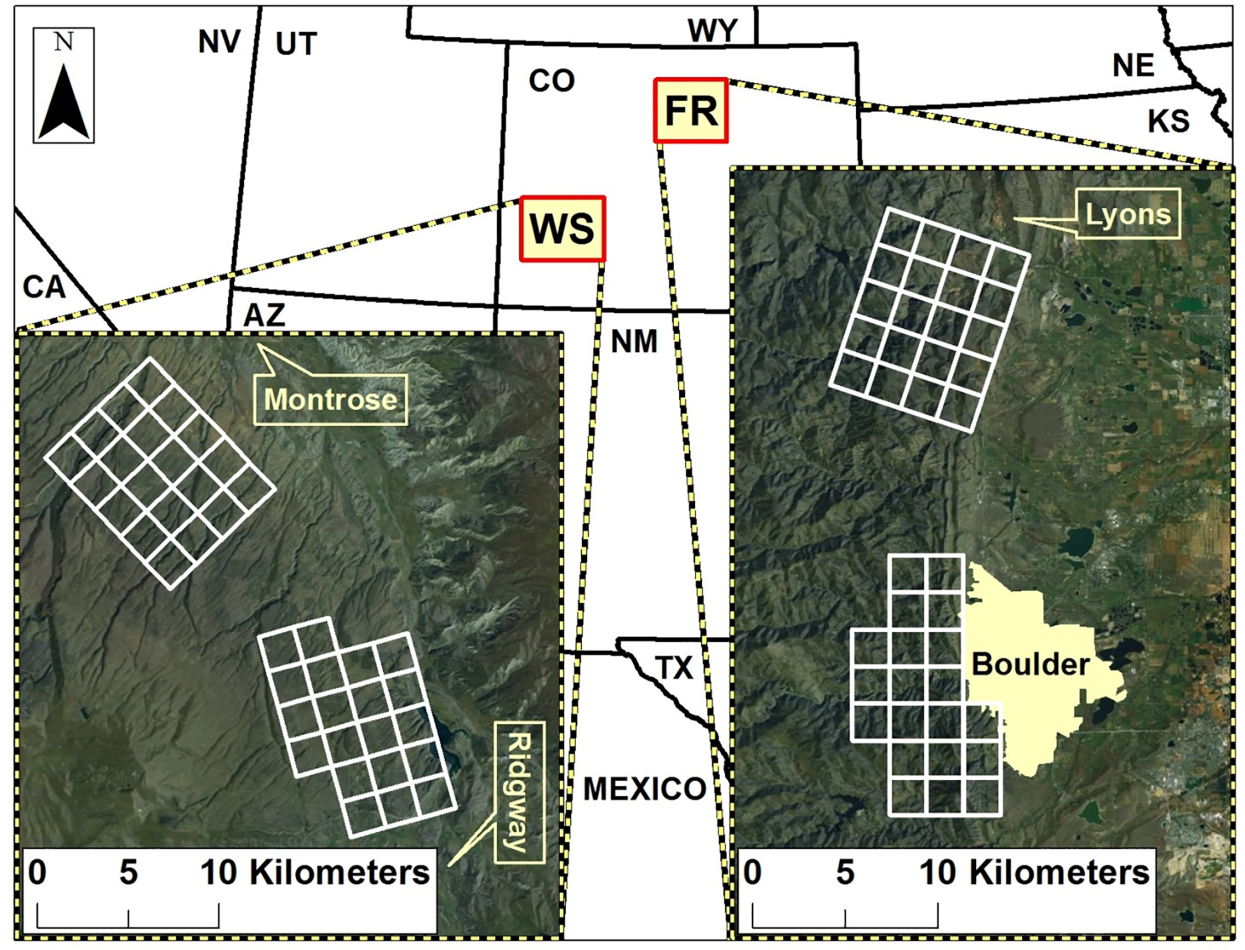
Locations of two study areas in Colorado, USA, which exhibited varying levels of urbanization, where bobcats and pumas were fit with telemetry collars. The more rural Western Slope (WS) was characterized by an exurban development south grid and a wildland north grid during 2009–2010. The more urbanized Front Range (FR) study area was characterized by a wildland-urban interface (WUI) south grid and wildland north grid during 2010–2012.

We conducted our research across two study areas in Colorado, USA that exhibited varying degrees of urbanization and human influence. In 2009–2010, we worked on the Western Slope (WS) of Colorado on the Uncompahgre Plateau near the towns of Montrose and Ridgway, which sampled areas of exurban development and wildland habitat (Fig 1). In 2010–2012, we worked on the more urbanized Front Range (FR) of Colorado, which sampled wildland-urban interface (WUI) habitat associated with the city of Boulder (population = 97,385, US Census Bureau 2010) and wildland habitat (Fig 1). Although uncommon, a small number of free-ranging domestic cats also occurred on our sampling grids on the WS and FR. See [65] for an expanded description of the study area.

### Animal capture and telemetry data

Bobcats were captured in black metal-wire cage traps during March 2009–2011 and immobilized through hand-injection of a combination of Ketamine (10.0 mg/kg) and Xylazine (1.0 mg/km), and Yohimbine (0.125 mg/km) was used to reverse Xylazine [66]. Adult-sized bobcats were fit with GPS collars (210–280 g, Telemetry Solutions, Concord, CA, USA). Pumas were captured from 2005–2011 with the use of hounds and baited cage traps, immobilized with Telazol (5.0–9.0 mg/kg), and fit with GPS collars (Lotek, Newmarket, Ontario, Canada; Northstar, King George, VA, USA; Vectronics, Berlin, Germany). See [65] for further details on animal capture methods and GPS telemetry. Methods for animal capture were approved by the Colorado State University Animal Care and Use Committee (11-2453A).

### Screening of pathogens in felids

**Fig 2:**
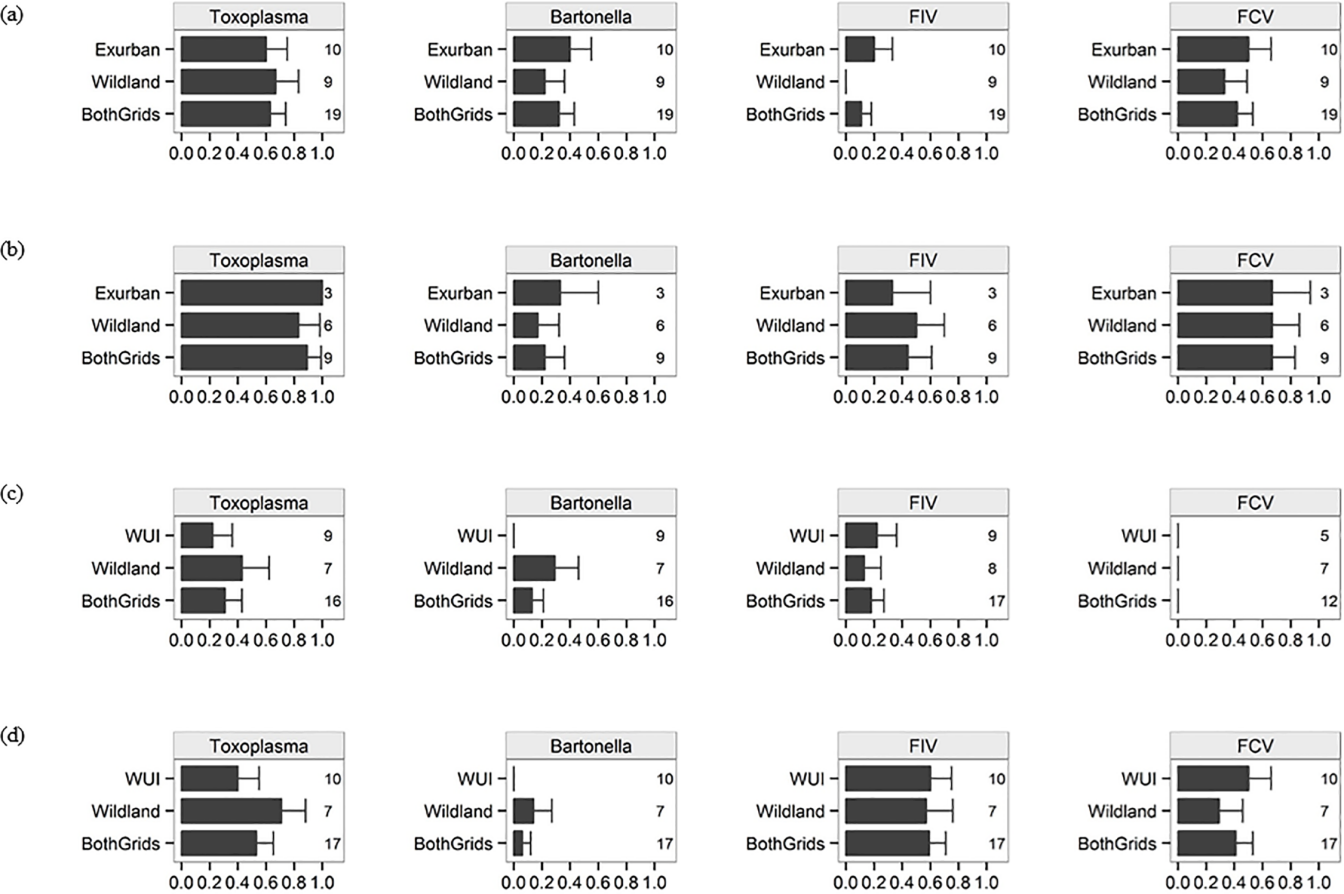
Prevalence of pathogens (estimates of seroprevalence and 1 standard error) for bobcats (a) and pumas (b) in exurban and wildland habitat on the Western Slope (WS) and for bobcats (c) and pumas (d) in wildland-urban interface (WUI) and wildland habitat on the Front Range (FR), Colorado. Sample sizes for the total number of animals screened for antibodies of each pathogen occur on the right side of each figure panel for the urbanized grid, wildland grid, and when both grids are combined.

We sampled 37 bobcats and 29 pumas on the WS and FR, although sample sizes varied across pathogens for groups of felids depending upon sample availability and quality (Fig 2; S1 Table). For each captured bobcat and puma, we collected blood (~10 mL) and saliva samples from immobilized animals for pathogen analysis. In the field, blood and serum samples were stored in ethylenedriaminetetraacetic acid (EDTA) and serum-sampling tubes that were immediately refrigerated. Samples were then transported within 24 hours to the CSU retrovirus research laboratory, where they were processed as previously reported [48, 61, 67]. To evaluate seroprevalence of pathogens (the detection of antibodies reacting against pathogen antigens used as a proxy for exposure to that pathogen), serum samples were analyzed for antibodies of Toxoplasma (enzyme-linked immunosorbent assay; ELISA), Bartonella (ELISA), FIV (Western Blot analysis), and FCV (ELISA) [48, 61]. Based on serum sample evaluations, individuals were classified as testing positive (i.e., antibodies against the pathogen detected = 1) or negative (i.e., antibodies against the pathogen not detected = 0). For further information about the accuracy of serological analyses for the four pathogens evaluated in our study, please see [48, 61, 67] and references therein.

### Pathogen characteristics and predictions

We hypothesized that multiple ecological factors would affect pathogen exposure in felid populations and focused our predictions on the expected relative strength that each mechanism would contribute to exposure of pathogens in felid populations.

*Toxoplasma gondii* (Toxoplasma) is a common pathogen in felids, with seroprevalence ranging from approximately 20–90% [61, 68–70]. Felids (domestic and wild cats) are the definitive host of Toxoplasma, in that infected animals excrete millions of oocysts into the environment over the course of approximately one week [71]. Felids can become infected by consuming infected prey, or less commonly, through direct environmental contamination by ingesting oocysts [54]. Once the infection is cleared, felids are assumed to be immune to reinfection and cease shedding oocysts into the environment, which can survive between several months and up to one year [60, 71]. Although Toxoplasma generally does not cause fitness effects in felids or humans, there are known behavioral impacts, and individuals that have a weakened immune system can experience complications [71, 72]. Environment and prey characteristics dictate patterns of Toxoplasma across the landscape (Table 1). Toxoplasma is associated with domestic cats, and thus can be more prevalent near areas of human residences, although the prevalence of Toxoplasma is predicted to vary across different forms of urbanization [60]. Low density urbanization, such as agricultural areas, can experience especially high prevalence of Toxoplasma due to an abundance of small mammals acting as intermediate hosts and sufficient predation of infected prey by domestic and wild felids. Some urban areas are predicted to exhibit lower prevalence of Toxoplasma due to fewer intermediate hosts and reduced numbers of predation events [54, 60, 73, 74]. Toxoplasma is reported to be more prevalent, and oocysts survival might be extended, in cool and wet years across regional to local areas [54, 73, 75]. Based on these relationships, we expect that animals inhabiting landscapes with greater plant productivity and moisture (i.e., as measured by Normalized difference vegetation index; NDVI; Table 2) would have a greater opportunity to be exposed to Toxoplasma (Table 1).

**Table 2:** Definitions of variables used in models evaluating pathogens in bobcats and pumas across a gradient of urbanization on the Western Slope (WS) and Front Range (FR) of Colorado. For further explanations of covariates see Methods.

Covariate	Category	Definition
Sex	Demographic	Male or female. For modeling, males = 0 and females = 1.
Age	Demographic	Continuous measure of age in years for adult-sized animals estimated based on dental characteristics and body size.
Space-use extent	Demographic	Spatial extent (km^2^) that an animal used based on space-use estimation of utilization distribution using the Brownian bridge movement model or kernel density methods. Because space-use extent is related to sex and age it was grouped with these variables.
Space-use overlap	Social (Intra- and Interspecific)	Overlap in space use between animals using the utilization distribution overlap index (UDOI) statistic [76].
Degree	Social (Intra- and Interspecific)	The number of neighbors an individual potentially interacted with based on overlap in space-use extents [77, 78].
In-strength	Social (Intra- and Interspecific)	The sum of space-use overlap values across all neighbors associated with an individual [79].
Equivalent social connectivity	Social (Intra- and Interspecific)	Equivalent social connectivity (ESC) among animals incorporates space-use overlap and extent [30]. This metric was based on equivalent connectivity [80], which was simplified to evaluate for an individual animal as follows: ESC_*i*_ = $\sqrt{\sum_{j=1}^{n}a_{i}a_{j}p_{ij}^{*}}$ where $a_{i}$ is the space-use extent for the focal animal *i*, $a_{j}$ are the spatial extents of space use for animals *j*, and $p_{ij}^{*}$ is the Bhattacharyya’s affinity (BA) statistic [76] used to define space-use overlap between animals *i* and *j* [30].
Amount of urbanization in space-use extent	Environment (Urban)	Human occurrence points (HOP; residences and structures) were digitized in ArcMap 10 and a kernel of 1000 m was fit over each HOP and kernels were summed to calculate human influence on the landscape [81]. An animal’s space-use extent was intersected with this layer and the amount of human influence was summed for each individual.
Grid	Environment (Urban)	Whether an animal was associated with exurban development or wildland grid on the WS or wildland-urban interface or wildland grid on the FR. For modeling, in each study area, urbanized grid = 0 and wildland grid = 1.
Amount of NDVI in space-use extent	Environment (Landscape)	The sum of the amount of Normalized difference vegetation index (NDVI; Pettorelli et al. 2005), which measures plant productivity and moisture across the landscape, within an animal’s extent of space use. NDVI was evaluated using eMODIS images (USGS August 2009 data on WS and August 2010 data on the FR).
NDVI per area of space-use extent	Environment (Landscape)	The amount of NDVI within an animal’s space-use extent divided by the area of space use for an individual.

*Bartonella spp*. (Bartonella) are a bacteria transmitted through flea, tick, and other arthropod vectors and can possibly lead to persistent or recurrent infection [64]. The bacteria are not generally deleterious to felid health, but can cause “cat scratch disease” in humans. There is a broad range in prevalence of Bartonella among bobcat populations (approximately 15–75%) and lower range for puma (approximately 10–40%) populations [61, 70, 82], where prevalence likely reflects each species’ exposure to arthropod vectors [61, 64]. Similar to Toxoplasma, Bartonella is associated with domestic cats (Table 1), which can potentially transmit the pathogen to wild felids primarily through flea vectors [64]. Bartonella is more prevalent in warm and humid climates where flea survival is increased [83, 84] and ticks can be associated with more moist environments [85–87]. Therefore, we might also expect that on finer spatial scales, animals with greater amounts of the landscape characterized by mesic environments (e.g., as measured by NDVI) within their extents of space use would have a greater likelihood of being exposed to vectors that harbor Bartonella (Table 1).

Feline immunodeficiency virus (FIV) is the felid equivalent of human immunodeficiency virus (HIV) and its prevalence varies widely across distinct bobcat and puma populations (approximately 20–60% prevalence) [28, 61, 62, 88]; however, it reportedly is not detected (prevalence of 0% for bobcats) in some populations [70, 89]. Each felid species is typically infected with a unique strain of FIV [90, 91], although cross-species transmission uncommonly occurs [62, 89, 90, 92]. FIV transmission events primarily occur through direct interactions (e.g., mating or aggressive encounters). Felids are infected with FIV throughout their lifetime; although most felids do not demonstrate clinical signs of infection, some individuals can potentially exhibit sublethal complications after many years of infection [91, 93]. Because FIV is transmitted through direct contacts, we expected greater prevalence in populations that exhibit more opportunity for interactions (Table 1). For example, populations that occur at higher densities would be expected to result in greater FIV prevalence [91]. Landscape pattern that is altered through urbanization can potentially influence population density and animal movement patterns, which could increase intra- and interspecific interactions [94] and thus the opportunity for FIV transmission [62, 95].

Feline calicivirus (FCV) is a widespread pathogen in felids, occurring at moderate levels in bobcat (prevalence ranging from 17–67%) [70] and puma (prevalence ranging from 17–56%) populations [88, 96, 97]. Although highly infectious and easily transmitted through direct contacts between animals, it typically only causes minor to moderate oral, ocular, and upper respiratory disease; however, more virulent outbreaks have occurred in domestic cats resulting in high mortality [98]. It is believed that felids can shed the virus for up to several months (and uncommonly throughout their lifetime) and although cats are believed to clear the virus, they can be reinfected with a related or novel viral variant of FCV [98]. The virus can be transmitted from adult females to their young, as well as among older animals. The prevalence of FCV increases with cat density [98], thus more contacts among animals increase the likelihood of being infected (Table 1). Although FCV can exhibit similar prevalence in male and female felids [99], we predicted that male bobcats and pumas might exhibit greater prevalence, compared to females, due to their larger home ranges and potential for increased contacts with other animals. Further, although FCV is probably most commonly transmitted via direct contacts between animals, the virus can persist in the environment (at least in clinical settings) for up to several weeks and thus potentially be transmitted indirectly (e.g., through urine and feces) [98], although it is unknown if this occurs in the natural environment. Because FCV is associated with domestic cats, we expect FCV prevalence to increase with the proximity to human residences where owned and feral cats reside and interactions with wild felids are most likely to occur [70] (Table 1). Lastly, within wild felid populations, the prevalence of FCV would be expected to follow similar predictions as presented for FIV above (Table 1).

### Modeling approach

#### Pathogen prevalence for felids across forms of urbanization

For each grid and study area, we evaluated bobcat and puma exposure to pathogens. We estimated the seroprevalence of each pathogen within felid populations across exurban development and wildland habitat on the WS and WUI and wildland habitat on the FR. Pathogen analysis was sometimes restricted by limited handling time of captured animals and sample quality that was occasionally inadequate for robust diagnostic testing. Therefore, the number of samples and animals evaluated was sometimes lower than the total number of animals captured in the field.

#### Evaluation of demographic, social, and environmental factors

Based on our predictions of pathogen prevalence in bobcat and puma (Table 1), we compared a suite of models [100, 101] evaluating demographic, social, and environmental characteristics for each pathogen in each felid population, which included 15 model sets (Tables 3 and 4). Sample size restricted the number of models that we could evaluate in some instances. Covariates were grouped into one of five categories: demographic, social intraspecific, social interspecific, environment urban, or environment landscape (Table 2). The demographic category included not only sex and age, but also space-use extent because this characteristic is related to both sex and age. Telemetry data were used to estimate space use of individuals by calculating the utilization distribution (UD) for felids that occurred on our sampling grids from June 2009 to June 2010 on the WS and September 2010 to September 2011 on the FR [30]. For animals fit with GPS collars (bobcats n = 37; pumas n = 25), UDs were estimated with the Brownian bridge movement model (BBMM) with the mkde package [102] in program R [103]. For pumas on the WS fit with VHF collars (n = 4), UDs were estimated with the kernel home range estimator using likelihood cross validation [104] in the Animal Space Use package [105]. We used the 99% cumulative probability of space use for all analyses.

**Table 3:** Variable importance values (VIV) for demographic, social (intraspecific and interspecific), and environmental (urban and landscape) categories for bobcats and pumas on the Western Slope (WS) and Front Range (FR) of Colorado, USA. VIV were used to assess the relative importance of groups of covariates in models evaluating pathogens in felid populations. A dash (i.e., -) indicates that models with this covariate could not be evaluated (see Methods).

			Demographic			Social Intraspecific		Social Interspecific		Environmental Urban		Environmental Landscape
Study Area	Species	Pathogen	Sex	Age	Space-Use Extent	Space-Use Overlap	Degree	Space-Use Overlap	Degree	Human Development	Grid	NDVI
WS	Bobcat	Toxoplasma	0.08	0.09	0.09	0.41	0.08	0.09	0.18	0.20	0.08	0.16
WS	Bobcat	Bartonella	0.10	0.13	0.14	0.15	0.13	0.33	0.11	0.20	0.16	0.11
WS	Bobcat	FIV	0.15	0.44	0.14	0.13	0.13	0.14	0.21	0.12	-	0.13
WS	Bobcat	FCV	0.04	0.05	0.42	0.55	0.05	0.16	0.17	0.06	0.08	0.04
WS	Puma	Toxoplasma	-	0.21	-	0.20	-	-	-	-	-	-
WS	Puma	Bartonella	-	-	-	0.13	-	0.12	0.15	0.12	0.12	0.18
WS	Puma	FIV	0.10	0.08	-	0.18	-	0.39	-	0.18	0.08	0.05
WS	Puma	FCV	0.10	-	-	0.26	-	0.13	-	0.19	0.07	0.11
FR	Bobcat	Toxoplasma	0.13	0.34	0.12	0.14	0.13	0.16	0.14	0.13	0.15	0.16
FR	Bobcat	Bartonella	-	0.09	0.03	0.19	0.05	0.70	0.04	0.08	-	0.13
FR	Bobcat	FIV	-	0.06	0.55	0.09	0.21	0.14	0.25	0.13	0.14	0.18
FR	Bobcat	FCV	-	-	-	-	-	-	-	-	-	-
FR	Puma	Toxoplasma	0.15	0.51	-	0.15	0.10	0.13	-	0.12	0.22	0.18
FR	Puma	Bartonella	-	0.15	-	0.30	0.18	0.23	-	0.15	-	0.15
FR	Puma	FIV	0.13	0.09	-	0.07	0.84	0.10	-	0.13	0.06	0.10
FR	Puma	FCV	0.25	0.19	-	0.11	0.13	0.18	-	0.10	0.15	0.37

**Table 4:** Model-averaged parameter estimates with associated standard errors for demographic, social (intraspecific and interspecific), and environmental (urban and landscape) categories for bobcats and pumas on the Western Slope (WS) and Front Range (FR) of Colorado, USA. A dash (i.e., -) indicates that models with this covariate could not be evaluated (see Methods).

			Demographic			Social Intraspecific		Social Interspecific		Environmental Urban		Environmental Landscape
Study Area	Species	Pathogen	Sex	Age	Space-Use Extent	Space-Use Overlap	Degree	Space-Use Overlap	Degree	Human Development	Grid	NDVI
WS	Bobcat	Toxoplasma	-0.21 (1.02)	-0.11 (0.27)	-0.10 (0.57)	1.06 (0.63)	0.00 (0.52)	0.00 (0.54)	-0.68 (0.68)	0.80 (0.77)	0.46 (1.21)	-0.50 (0.56)
WS	Bobcat	Bartonella	-0.31 (1.07)	0.16 (0.25)	0.42 (0.63)	-0.45 (0.62)	0.57 (0.65)	1.08 (0.77)	-0.31 (0.59)	0.74 (0.57)	-1.10 (1.28)	-0.33 (0.64)
WS	Bobcat	FIV	-1.34 (2.53)	0.71 (0.47)	-0.53 (1.37)	-0.26 (1.15)	-0.31 (0.81)	-0.75 (1.38)	1.50 (1.52)	-0.36 (1.13)	-	0.56 (0.88)
WS	Bobcat	FCV	-0.13 (1.04)	0.08 (0.25)	-1.72 (1.24)	-1.72 (1.26)	0.26 (0.63)	-1.03 (0.95)	-1.53 (1.53)	-0.52 (0.73)	-1.22 (1.35)	0.10 (0.54)
WS	Puma	Toxoplasma	-	1.23 (2.08)	-	1.57 (2.32)	-	-	-	-	-	-
WS	Puma	Bartonella	-	-	-	-0.71 (1.06)	-	0.44 (0.91)	-1.02 (1.59)	-0.43 (1.07)	-0.87 (1.83)	-1.78 (1.96)
WS	Puma	FIV	-0.90 (1.87)	0.19 (0.73)	-	1.50 (1.70)	-	-2.81 (2.42)	-	1.41 (1.11)	0.16 (2.01)	-0.32 (1.21)
WS	Puma	FCV	0.08 (1.66)	-	-	1.52 (1.64)	-	0.27 (0.82)	-	1.14 (1.28)	-1.70 (3.71)	0.03 (1.22)
FR	Bobcat	Toxoplasma	0.55 (1.33)	0.57 (0.44)	-0.03 (0.65)	0.73 (0.88)	-0.22 (0.67)	0.57 (0.75)	-0.49 (0.67)	0.27 (0.68)	1.10 (1.23)	-0.53 (0.74)
FR	Bobcat	Bartonella	-	-1.18 (1.24)	0.76 (1.08)	1.71 (2.61)	1.22 (1.73)	5.46 (4.81)	0.98 (1.11)	-5.02 (4.48)	-	-0.51 (2.29)
FR	Bobcat	FIV	-	-0.05 (0.47)	2.53 (2.34)	0.15 (1.02)	-1.58 (1.55)	-0.83 (0.86)	-3.02 (3.33)	-1.03 (1.97)	-3.52 (4.72)	-2.53 (3.40)
FR	Bobcat	FCV	-	-	-	-	-	-	-	-	-	-
FR	Puma	Toxoplasma	1.08 (1.26)	1.08 (0.69)	-	0.65 (0.76)	-0.14 (0.60)	0.47 (0.63)	-	-0.43 (0.82)	1.65 (1.24)	0.87 (1.02)
FR	Puma	Bartonella	-	0.34 (1.32)	-	2.24 (3.03)	1.30 (2.80)	-2.06 (2.42)	-	-0.39 (1.78)	-	0.66 (1.96)
FR	Puma	FIV	1.44 (1.36)	-0.30 (0.44)	-	0.25 (0.74)	-4.05 (2.79)	0.52 (0.77)	-	0.93 (1.28)	-0.33 (1.28)	0.65 (1.08)
FR	Puma	FCV	1.73 (1.31)	0.47 (0.44)	-	0.23 (0.64)	0.49 (0.76)	0.67 (0.61)	-	0.22 (0.91)	-0.84 (1.17)	3.57 (3.61)

For social interactions, to evaluate the opportunity for direct and indirect contacts between individuals, we estimated *space-use overlap* among animals [79, 106, 107]; this information was used to estimate *degree*, *in-strength*, and *equivalent social connectivity* for intra- and interspecific social interactions (Table 2) [30]. We defined social interactions as potential direct or indirect contacts occurring between animals, including both intra- and interspecific interactions. Although social behavior is most commonly associated with intraspecific interactions (e.g., [108, 109]), social interactions can also occur between species, for example in the context of interspecific dominance relationships [110, 111].

We also evaluated several environmental covariates, including the *amount of urbanization in space-use extent*, *grid*, and NDVI (Table 1). Each continuous covariate was standardized by subtracting the sample mean from the input variable values and dividing by the standard deviation [112]. Covariates were evaluated for collinearity using Pearson’s correlation and considered correlated if r > 0.7; amount of NDVI in *space-use extent* and intra- and interspecific *in-strength* and *effective social connectivity* were highly correlated with multiple covariates and were subsequently excluded from analyses. In addition, *space-use extent* for WS and FR pumas and *interspecific-degree* for FR pumas were highly correlated with multiple covariates and were excluded from analyses. For further explanations about how social interaction and urban covariates were calculated see Table 2 and [30].

Using logistic regression in R (i.e., glm with binomial logit link [103]), we evaluated model sets that were comprised of all possible combinations of univariate covariates and pairwise comparisons (56 total models) for each species in each study area and ranked models using Akaike’s Information Criteria corrected for small sample size (AIC_c_) [101]. We evaluated all possible combinations of covariates in models [113] with up to 2 covariates based on sample size recommendations of evaluating 1 covariate per 5–10 observations [114]. Larger sample sizes would allow the evaluation of all variable combinations. To evaluate the relative importance of variables in models, we calculated variable importance values (VIV) and model-averaged parameter estimates across models in which they occurred [101, 115]. Likely due to either relatively low or high prevalence of the pathogen in logistic regression models, coupled with relatively low sample sizes, models sometimes failed to converge; these models generally had little support in our data sets (i.e., AIC weight ≤ 0.01) and were removed to calculate VIV and model-averaged parameter estimates. To evaluate which covariates were supported in our model sets, we first identified covariates based on whether they occurred in models that performed better than the intercept-only model. We then evaluated their VIV in model sets and the direction of their model-averaged parameter estimates.

## Results

Both felids used areas in close proximity to human residences in exurban development and along the wildland-urban interface. Seroprevalence for pathogens was evaluated for 71%–100% of sampled individuals within populations (Fig 2), with the proportion of the sampled population screened based on sample quantity and quality. Although seroprevalence for some pathogens varied between grids (Fig 2), we did not find support for a statistical difference in seroprevalance between urbanized and wildland grids based on the covariate Grid not occurring in top models (S2–S16 Tables) and when evaluating VIVs (≤0.22; Table 3) and model-averaged parameter estimates (95% confidence intervals overlapped 0; Table 4).

### Effects of demographic, social, and environmental factors

#### Demographic factors

As predicted for some pathogens, individuals were more likely to be exposed with increasing age; this covariate occurred in the top-ranked models for FIV in WS bobcats (S3 Table) and Toxoplasma in FR bobcats (S10 Table) and FR pumas (S13 Table). VIV in these instances ranged from 0.34 to 0.51 (Table 3), and the model-averaged parameter estimates indicated a positive trend, although 95% confidence intervals overlapped 0, between exposure and age (WS bobcats FIV: β = 0.71, se = 0.47; FR bobcats Toxoplasma: β = 0.57, se = 0.44; FR pumas Toxoplasma: β = 1.08, se = 0.69; Table 4). In support of our predictions, *space-use extent* for FIV in FR bobcats occurred in the three top-ranked models (S12 Table) with a VIV = 0.55 (Table 3) and a positive trend with exposure (β = 2.54, se = 2.34; Table 4). However, counter to predictions, *space use extent* was negatively related to FCV for WS bobcats (β = -1.72, se = 1.24; Table 4); this covariate occurred in top-ranked models (S5 Table) and had a VIV = 0.42 (Table 3).

#### Social factors

As predicted, WS bobcats appeared more likely to be exposed to Toxoplasma with increasing *intraspecific space-use overlap* (β = 1.06, se = 0.63; Table 4); this covariate occurred in the top-ranked model (S2 Table) with a VIV = 0.41 (Table 3). However, counter to predictions for directly transmitted pathogens, exposure to FCV for WS bobcats and FIV for FR pumas appeared negatively related to *intraspecific space-use overlap* (β = -1.72, se = 1.26) and *intraspecific degree* (β = -4.05, se = 2.79), respectively (Table 4); these covariates occurred in the suite of top-ranked models (S4 and S14 Tables, respectively) with VIVs of 0.55 and 0.84, respectively (Table 3). Exposure to Bartonella appeared to be positively related to *interspecific space-over overlap* for WS (β = 1.08, se = 0.77) and FR (β = 5.46, se = 4.81) bobcats (Table 4); this covariate occurred in the top-ranked models for each model set (S3 and S11 Tables, respectively) with VIVs of 0.33 and 0.70, respectively (Table 3).

#### Environmental factors

In contrast to predictions, the environmental covariates evaluating urban and landscape features were not well supported in our models (Tables 3 and 4; S2–S16 Tables). In general, environmental covariates did not occur in top-ranked models, with the exception of NDVI for FCV in FR pumas (S16 Table), which exhibited a VIV of 0.37 (Table 3) and was positively related to FCV exposure (β = 3.57 and se = 3.61; Table 4).

## Discussion

Demographic, social, and environmental factors varied in their association with pathogen exposure in bobcat and puma populations. As expected, demographic factors helped explain exposure to some pathogens in our study. Specifically, age appeared to be positively related to exposure to pathogens that are both environmentally transmitted (Toxoplasma) and directly transmitted between animals (FIV), consistent with our predictions and other studies of felid populations [28, 48, 61]. We predicted that animals with greater extents of space use would be more likely to interact with other individuals and greater extents of the landscape, leading to a greater probability of pathogen transmission [30]. However, we found weak and equivocal support for the effects of space-use extent on pathogen exposure. Although these results were counter to our predictions, other factors related to space use might be important considerations for explaining pathogen characteristics, including social interactions and status, as explained below.

Social interactions appeared to influence exposure to some pathogens via indirect and direct means of transmission. As predicted, as *intraspecific space-use overlap* increased within bobcat populations, animals were more likely to be exposed to Toxoplasma. Felids may increase marking behavior along territorial boundaries and in areas of sympatry [34]. Because felids are the definitive host of Toxoplasma and excrete oocysts into the environment via scats [54, 60, 71], areas of shared space use would likely exhibit increased concentrations of Toxoplasma and elevated levels of Toxoplasma in prey. In addition, Toxoplasma likely is present at high concentrations at felid marking locations where animals repeatedly scat and urinate [54]; because animals revisit these sites and investigate the markings of other animals (both within and between species), animals could experience a relatively high chance of being exposed to Toxoplasma through environmental contamination.

Both *space-use overlap* and *degree* are positively correlated with *space-use extent*, which itself is related to gender and the behavior of resident or transient animals [30]. In wild felids, females and residents generally express smaller extents of space-use than males and transients, which are associated with increased movement extents with less pronounced site fidelity [34]. Transients, which often are younger animals without a defined home range, could potentially exhibit two different patterns of behavior that would influence pathogen characteristics. They might either (1) exhibit reduced interactions due to fewer opportunities to mate and defend a territory [34], which could explain the negative correlation between FCV exposure and space use extent in WS bobcats, or (2) exhibit increased interactions when attempting to establish a home range. Because these patterns have not been well evaluated in wild felid populations, the effect of social status and behavior on contact and disease transmission is in need of further study.

Counter to studies in other systems (e.g., [40, 62]), our data did not indicate that the interspecific factors that we evaluated strongly influenced cross-species transmission of our four target pathogens between bobcats and pumas. However, *interspecific space-use overlap* did appear to increase exposure to Bartonella in bobcats. This is possibly related to both bobcats and pumas using similar habitat with elevated levels of Bartonella, such as areas associated with domestic cats or other sources (e.g., vectors such as fleas or ticks) of the pathogen [64]. It is also possible that bobcats acquired vectors that transmitted this pathogen from pumas.

Environmental variables in our models appeared to have the least support in explaining exposure of the four pathogens we evaluated in felid populations. Neither the amount nor the type of urbanization with which animals were associated predicted exposure to pathogens. Different forms of urbanization (i.e., exurban vs urban) can alter prey and domestic cat populations, both of which could be important factors for transmitting pathogens to wild felids [54, 60]. Additional research, however, is necessary to quantify how these factors varied across the landscape in our study system, especially in relation to feral cat populations and seroprevalence of Toxoplasma in small mammal populations. Further, although directly-transmitted pathogens might be more likely to be transmitted in urbanized landscapes due to increased interactions of felids [62], other research in our study areas reported similar amounts of *interspecific space-use overlap* and potential contact rates in felids across broad scales between urbanized and wildland habitat [30]. Importantly, increased interaction opportunities at fine temporal scales can occur in urbanized landscapes [116], which is predicted to increase cross-species pathogen transmission in urban areas. For pathogens that are associated with specific vectors (e.g., ectoparasites) or intermediate hosts (e.g., small mammals), it could be useful to create predictive maps of habitat association for these organisms [117–124] and use this information as covariates in models. Additionally, other environmental factors, such as soil characteristics, can be associated with the risk of disease [125].

The relatively small number of individuals screened for pathogen exposure reduced our power to detect differences in this parameter in relation to covariates [126]. Although we used GPS telemetry to track a comparatively large proportion of the population of bobcats and pumas occurring concurrently within our study areas, studies with greater sample sizes are needed to further understand the effects of demographic, social, and environmental factors on pathogen characteristics in wildlife populations. In addition, variation in diagnostic assay sensitivity and specificity can result in false negative or false positive assignments, further increasing uncertainty [127, 128]. In our study, although we detected low seroprevalence of FIV in bobcats [61], FIV PCR analyses for the same individuals failed to detect the presence of FIV [89], indicating uncertainty that FIV infection occurred in this cohort. Further, the spatial scale of analysis can strongly influence inference [129]. Although our study did not find strong results at relatively fine spatial scales, broad-scale analyses of pathogens might better explain patterns [48, 126]. At fine scales, patterns of pathogen exposure might appear homogenous, but at broader spatial scales patterns may become more heterogeneous. For example, some vectors, such as ticks, demonstrate a gradient of population densities across their geographic range, where they are most abundant at the interior of their range and decrease in density by 1–2 orders of magnitude at the edge of their range [130]; such patterns could affect the opportunity for animals to be exposed to pathogens.

Although many studies focus on single pathogen characteristics within a single species, pathogen spillover between species is increasingly recognized as important to understanding disease epidemics in wildlife populations [1, 126, 131]. Spillover events may be occasional, followed by self-sustaining transmission within the new host species. For example, although each felid species typically harbors a unique strain of FIV [90], transmission of species specific strains of FIV has occurred between pumas and bobcats [62, 132]. In other cases, spillover may not occur between species due to the specificity between the pathogen and host. Due to the lack of genotypic information to assess pathogen spillover in a multihost, multipathogen system additional investigation are warranted to further investigate this important topic in relation to anthropogenic landscape change.

In addition to providing insight on pathogen exposure in wild felids, our study provides an approach for evaluating how demographic, social, and environmental factors influence disease dynamics in animal populations, which allows for the comparison and evaluation of the relative strength among multiple mechanisms and hypotheses. Future work applying this strategy will be necessary to gain a better understanding about how ecological mechanisms influence pathogen exposure and transmission, with important implications for the conservation of animal populations.

## Supporting information

S1 Table Sample sizes for pathogen samples of bobcats and pumas for exurban and wildland habitat on the Western Slope (WS) in 2009 and wildland-urban interface (WUI) and wildland habitat on the Front Range (FR) in 2010–2011, Colorado, USA. (DOC)

S2 Table Model results for Toxoplasma in bobcats on the Western Slope of Colorado, USA. (DOC)

S3 Table Model results for Bartonella in bobcats on the Western Slope of Colorado, USA. (DOC)

S4 Table Model results for FIV in bobcats on the Western Slope of Colorado, USA. (DOC)

S5 Table Model results for Calicivirus in bobcats on the Western Slope of Colorado, USA. (DOC)

S6 Table Model results for Toxoplasma in pumas on the Western Slope of Colorado, USA. (DOC)

S7 Table Model results for Bartonella in pumas on the Western Slope of Colorado, USA. (DOC)

S8 Table Model results for FIV in pumas on the Western Slope of Colorado, USA. (DOC)

S9 Table Model results for Calicivirus in pumas on the Western Slope of Colorado, USA. (DOC)

S10 Table Model results for Toxoplasma in bobcats on the Front Range of Colorado, USA. (DOC)

S11 Table Model results for Bartonella in bobcats on the Front Range of Colorado, USA. (DOC)

S12 Table Model results for FIV in bobcats on the Front Range of Colorado, USA. (DOC)

S13 Table Model results for Toxoplasma in pumas on the Front Range of Colorado, USA. (DOC)

S14 Table Model results for Bartonella in pumas on the Front Range of Colorado, USA. (DOC)

S15 Table Model results for FIV in pumas on the Front Range of Colorado, USA. (DOC)

S16 Table Model results for Calicivirus in pumas on the Front Range of Colorado, USA. (DOC)

S17 Table Data used to evaluate Toxoplasma seroprevalence in bobcats on the Western Slope of Colorado, USA. (TXT)

S18 Table Data used to evaluate Bartonella seroprevalence in bobcats on the Western Slope of Colorado, USA. (TXT)

S19 Table Data used to evaluate FIV seroprevalence in bobcats on the Western Slope of Colorado, USA. (TXT)

S20 Table Data used to evaluate Calicivirus seroprevalence in bobcats on the Western Slope of Colorado, USA. (TXT)

S21 Table Data used to evaluate Toxoplasma seroprevalence in pumas on the Western Slope of Colorado, USA. (TXT)

S22 Table Data used to evaluate Bartonella seroprevalence in pumas on the Western Slope of Colorado, USA. (TXT)

S23 Table Data used to evaluate FIV seroprevalence in pumas on the Western Slope of Colorado, USA. (TXT)

S24 Table Data used to evaluate Calicivirus seroprevalence in pumas on the Western Slope of Colorado, USA. (TXT)

S25 Table Data used to evaluate Toxoplasma seroprevalence in bobcats on the Front Range of Colorado, USA. (TXT)

S26 Table Data used to evaluate Bartonella seroprevalence in bobcats on the Front Range of Colorado, USA. (TXT)

S27 Table Data used to evaluate FIV seroprevalence in bobcats on the Front Range of Colorado, USA. (TXT)

S28 Table Data used to evaluate Toxoplasma seroprevalence in pumas on the Front Range of Colorado, USA. (TXT)

S29 Table Data used to evaluate Bartonella seroprevalence in pumas on the Front Range of Colorado, USA. (TXT)

S30 Table Data used to evaluate FIV seroprevalence in pumas on the Front Range of Colorado, USA. (TXT)

S31 Table Data used to evaluate Calicivirus seroprevalence in pumas on the Front Range of Colorado, USA. (TXT)
