# Supplementary material for: The effects of demographic, social, and environmental characteristics on pathogen prevalence in wild felids across a gradient of urbanization
Source: PLoS One. 2017 Nov 9;12(11):e0187035. doi: 10.1371/journal.pone.0187035 (PMC5679604; doi:10.1371/journal.pone.0187035)
Supplement: S1 Table — (DOC) [file pone.0187035.s001.doc]

**S1 Table**. Sample sizes for pathogen samples of bobcats and pumas for exurban and wildland habitat on the Western Slope (WS) in 2009 and wildland-urban interface (WUI) and wildland habitat on the Front Range (FR) in 2010 - 2011, Colorado, USA.
