# Supplementary material for: The effects of demographic, social, and environmental characteristics on pathogen prevalence in wild felids across a gradient of urbanization
Source: PLoS One. 2017 Nov 9;12(11):e0187035. doi: 10.1371/journal.pone.0187035 (PMC5679604; doi:10.1371/journal.pone.0187035)
Supplement: S6 Table — (DOC) [file pone.0187035.s006.doc]

**S6 Table**. Model results for Toxoplasma in pumas on the Western Slope of Colorado, USA.
